# Supplementary material for: Using a Dynamic Model to Estimate the Cost-Effectiveness of HPV Vaccination in Iran
Source: Vaccines (Basel). 2024 Apr 18;12(4):438. doi: 10.3390/vaccines12040438 (PMC11054652; doi:10.3390/vaccines12040438)
Supplement: Supplementary file 1 [file vaccines-12-00438-s001.zip › vaccines-2924910-supplementary.pdf]

Supplementary File S1 – Model parameters

1. Parameters sources

Parameter	Description	
**Demographics**		
Pop_frac	Population composition	[1]
Birth	Normal birthrate	[2]
Mortality	Background mortality rate	[3]
**Contact matrix**		[4,5] and assumptions
P_CC	Probability on cervical cancer after HPV	Calibration
I_dur	Duration of infectious period (years)	[6]
Lat_CC	CC latency (years)	[7]
**Vaccination and fatality**		
Vac_Uptake	Vaccination uptake	Assumption
Vac_Eff_Inf	Vaccination effectiveness on infection	[8]
f_CCT	Fraction of cervical cancer patients treated	[9]
CCT_D	Fraction of treated cervical cancer patients to die	[10]
CCU_D	Fraction of untreated cervical cancer patients to die	Assumption
CCT_dur	Duration with cervical cancer treated	[10]
CCU_dur	Duration with cervical cancer untreated	Assumption
Im_dur	Immunity duration (years)	Assumption
**Economics**		
Rf	Financial discount rate	[11]
Re	Effect QALY discount rate	[11]
CoV	Cost of vaccination	[12]
Utility	Utility healthy	[13]
Ut_CC	Relative utility cervical cancer	[12,14]
Ut_UT	Relative utility untreated vs treated	Assumption
CoCaseCCt	Cost per CC case treated	[12]

2. Parameter Values—Men

Parameter	Description	0 (0-9)	1 (10-14)	2 (15-19)	3 (20-24)	4 (25-29)	5 (30-34)	6 (35-39)	7 (40-44)	8 (45-49)	9 (50-54)	10 (55-59)	11 (60-64)	12 (65-69)	13 (70-74)	14 (75-79)	15 (80+)
**Demographics**																	
Pop_frac		0.084	0.038	0.033	0.032	0.039	0.050	0.051	0.041	0.032	0.028	0.023	0.020	0.015	0.010	0.005	0.004
Birth	Normal birthrate																
Mortality	Background Mortality Rate	0.0030223	0.0002794	0.0007386	0.0009734	0.0009185	0.0009798	0.0011539	0.0015126	0.0021391	0.0034809	0.005612	0.0086434	0.0129964	0.0201837	0.0354535	0.0849798
**Sexual partners matrix**																	
C_M_1	Contacts of A with Age group Partn	-	0	0	0	0	0	0	0	0	0	0	0	0	0	0	0
C_M_2		0	0	0	0	0	0	0	0	0	0	0	0	0	0	0	0
C_M_3		0	0	0	0	0	0	0	0	0	0	0	0	0	0	0	0
C_M_4		0	0	0	0	0	0	0	0	0	0	0	0	0	0	0	0
C_M_5		0	0	0	0	0	0	0	0	0	0	0	0	0	0	0	0
C_M_6		0	0	0	0	0	0	0	0	0	0	0	0	0	0	0	0
C_M_7		0	0	0	0	0	0	0	0	0	0	0	0	0	0	0	0
C_M_8		0	0	0	0	0	0	0	0	0	0	0	0	0	0	0	0
C_M_9		0	0	0	0	0	0	0	0	0	0	0	0	0	0	0	0
C_M_10		0	0	0	0	0	0	0	0	0	0	0	0	0	0	0	0
C_M_11		0	0	0	0	0	0	0	0	0	0	0	0	0	0	0	0
C_M_12		0	0	0	0	0	0	0	0	0	0	0	0	0	0	0	0
C_M_13		0	0	0	0	0	0	0	0	0	0	0	0	0	0	0	0
C_M_14		0	0	0	0	0	0	0	0	0	0	0	0	0	0	0	0
C_M_15		0	0	0	0	0	0	0	0	0	0	0	0	0	0	0	0
C_M_16		0	0	0	0	0	0	0	0	0	0	0	0	0	0	0	0
C_F_1	Contacts of A with Age group Partn	0	0	0	0	0	0	0	0	0	0	0	0	0	0	0	0
C_F_2		0	3.5	0	0	0	0	0	0	0	0	0	0	0	0	0	0
C_F_3		0	11.403	11.17494	0.22806	0	0	0	0	0	0	0	0	0	0	0	0
C_F_4		0	0	14.13972	27.3672	4.10508	0	0	0	0	0	0	0	0	0	0	0
C_F_5		0	0	0	11.403	26.91108	6.8418	0	0	0	0	0	0	0	0	0	0
C_F_6		0	0	0	0	6.8418	26.91108	10.94688	0.45612	0	0	0	0	0	0	0	0
C_F_7		0	0	0	0	0	6.8418	26.91108	10.94688	0.45612	0	0	0	0	0	0	0
C_F_8		0	0	0	0	0	0	6.8418	26.91108	10.94688	0.45612	0	0	0	0	0	0
C_F_9		0	0	0	0	0	0	0	6.8418	26.91108	10.94688	0.45612	0	0	0	0	0
C_F_10		0	0	0	0	0	0	0	0	6.8418	26.91108	10.94688	0.45612	0	0	0	0
C_F_11		0	0	0	0	0	0	0	0	0	6.8418	26.91108	10.94688	0.45612	0	0	0
C_F_12		0	0	0	0	0	0	0	0	0	0	3.0681	12.06786	4.90896	0.20454	0	0
C_F_13		0	0	0	0	0	0	0	0	0	0	0	3.0681	12.06786	4.90896	0.20454	0
C_F_14		0	0	0	0	0	0	0	0	0	0	0	0	2.0804	8.1829067	3.32864	0.1386933
C_F_15		0	0	0	0	0	0	0	0	0	0	0	0	0	1.0927	4.2979533	1.74832
C_F_16		0	0	0	0	0	0	0	0	0	0	0	0	0	0	0.105	0.413
P_CC	Probability on cervical cancer after I	**0**	**0**	**0**	**0**	**0**	**0**	**0**	**0**	**0**	**0**	**0**	**0**	**0**	**0**	**0**	**0**
I_dur	Duration of infectious period (years	**1.5**															
Lat_CC	CC Latency (years)	20	20	20	20	20	20	20	20	20	20	20	20	20	20	20	20
**Vaccination and fatality**																	
Vac_Uptake	Vaccination uptake	**0**	**0**	**0**	**0**	**0**	**0**	**0**	**0**	**0**	**0**	**0**	**0**	**0**	**0**	**0**	**0**
Vac_Eff_Inf	Vaccination effectiveness on infecti	**0.99**	**0.99**	**0.99**	**0.99**	**0.99**	**0.99**	**0.99**	**0.99**	**0.99**	**0.99**	**0.99**	**0.99**	**0.99**	**0.99**	**0.99**	**0.99**
f_CCT	Fraction of Cervical Cancer patients	0	0	0	0	0	0	0	0	0	0	0	0	0	0	0	0
CCT_D	Fraction of treated Cervical Cancer	0	0	0	0	0	0	0	0	0	0	0	0	0	0	0	0
CCU_D	Fraction of untreated Cervical Canc	0	0	0	0	0	0	0	0	0	0	0	0	0	0	0	0
CCT_dur	Duration with cervical cancer treate	1	1	1	1	1	1	1	1	1	1	1	1	1	1	1	1
CCU_dur	Duration with cervical cancer untrea	1	1	1	1	1	1	1	1	1	1	1	1	1	1	1	1
Im_dur	Immunity duration (years)	10															
**Economics**																	
Rf	Financial discount rate	3.5%															
Re	effect QALY discount rate	3.5%															
CoV	Cost of Vaccination	152.00															
CoD	Cost of premature death																
CoPLL	Cost of productivity loss																
Utility	Utility healthy	1	1	1	0.99	0.98	0.97	0.96	0.95	0.93	0.91	0.89	0.87	0.84	0.81	0.78	0.75
Ut_CC	Rel Utility cervical cancer	0.62															
Ut_UT	Relative utility untreated vs treated	0.5															
CoCaseCCt	Cost per CC case treated	26250															

3. Parameter Values—Women

		0 (0-9)	1 (10-14)	2 (15-19)	3 (20-24)	4 (25-29)	5 (30-34)	6 (35-39)	7 (40-44)	8 (45-49)	9 (50-54)	10 (55-59)	11 (60-64)	12 (65-69)	13 (70-74)	14 (75-79)	15 (80+)
**Demographics**																	
Pop_frac		0.080	0.036	0.032	0.031	0.038	0.048	0.050	0.040	0.031	0.027	0.023	0.019	0.015	0.011	0.007	0.007
Birth	Normal birthrate (set also by forecast sheet)																
Mortality	Background Mortality Rate	0.0027194	0.0002121	0.0003177	0.0003439	0.0003883	0.0004366	0.0005375	0.0007434	0.001126	0.0018205	0.0030565	0.0054458	0.0090769	0.0158956	0.0312893	0.0870697
AgeWidth	Width in years of the age group	10	5	5	5	5	5	5	5	5	5	5	5	5	5	5	5
**Sexual partners matrix**																	
C_M_1	Contacts of A with Age group Partn	0	0	0	0	0	0	0	0	0	0	0	0	0	0	0	0
C_M_2		0	4	13.032	0	0	0	0	0	0	0	0	0	0	0	0	0
C_M_3		0	0	12.77136	16.15968	0	0	0	0	0	0	0	0	0	0	0	0
C_M_4		0	0	0.26064	31.2768	13.032	0	0	0	0	0	0	0	0	0	0	0
C_M_5		0	0	0	4.69152	30.75552	7.8192	0	0	0	0	0	0	0	0	0	0
C_M_6		0	0	0	0	7.8192	30.75552	7.8192	0	0	0	0	0	0	0	0	0
C_M_7		0	0	0	0	0	12.51072	30.75552	7.8192	0	0	0	0	0	0	0	0
C_M_8		0	0	0	0	0	0.52128	12.51072	30.75552	7.8192	0	0	0	0	0	0	0
C_M_9		0	0	0	0	0	0	0.52128	12.51072	30.75552	7.8192	0	0	0	0	0	0
C_M_10		0	0	0	0	0	0	0	0.52128	12.51072	30.75552	7.8192	0	0	0	0	0
C_M_11		0	0	0	0	0	0	0	0	0.52128	12.51072	30.75552	3.5064	0	0	0	0
C_M_12		0	0	0	0	0	0	0	0	0	0.52128	12.51072	13.79184	3.5064	0	0	0
C_M_13		0	0	0	0	0	0	0	0	0	0	0.52128	5.61024	13.79184	2.3776	0	0
C_M_14		0	0	0	0	0	0	0	0	0	0	0	0.23376	5.61024	9.3518933	1.2488	0
C_M_15		0	0	0	0	0	0	0	0	0	0	0	0	0.23376	3.80416	4.9119467	0.12
C_M_16		0	0	0	0	0	0	0	0	0	0	0	0	0	0.1585067	1.99808	0.472
C_F_1	Contacts of A with Age group Partn	0	0	0	0	0	0	0	0	0	0	0	0	0	0	0	0
C_F_2		0	0	0	0	0	0	0	0	0	0	0	0	0	0	0	0
C_F_3		0	0	0	0	0	0	0	0	0	0	0	0	0	0	0	0
C_F_4		0	0	0	0	0	0	0	0	0	0	0	0	0	0	0	0
C_F_5		0	0	0	0	0	0	0	0	0	0	0	0	0	0	0	0
C_F_6		0	0	0	0	0	0	0	0	0	0	0	0	0	0	0	0
C_F_7		0	0	0	0	0	0	0	0	0	0	0	0	0	0	0	0
C_F_8		0	0	0	0	0	0	0	0	0	0	0	0	0	0	0	0
C_F_9		0	0	0	0	0	0	0	0	0	0	0	0	0	0	0	0
C_F_10		0	0	0	0	0	0	0	0	0	0	0	0	0	0	0	0
C_F_11		0	0	0	0	0	0	0	0	0	0	0	0	0	0	0	0
C_F_12		0	0	0	0	0	0	0	0	0	0	0	0	0	0	0	0
C_F_13		0	0	0	0	0	0	0	0	0	0	0	0	0	0	0	0
C_F_14		0	0	0	0	0	0	0	0	0	0	0	0	0	0	0	0
C_F_15		0	0	0	0	0	0	0	0	0	0	0	0	0	0	0	0
C_F_16		0	0	0	0	0	0	0	0	0	0	0	0	0	0	0	0
P_CC	Probability on cervical cancer after	**0.001125**	**0.001125**	**0.001125**	**0.001125**	**0.001125**	**0.001125**	**0.001125**	**0.001125**	**0.001125**	**0.001125**	**0.001125**	**0.001125**	**0.001125**	**0.001125**	**0.001125**	**0.001125**
I_dur	Duration of infectious period (years)																
Lat_CC	CC Latency (years)	10	10	10	10	10	10	10	10	10	10	10	10	10	10	10	10
**Vaccination and fatality**																	
Vac_Uptake	Vaccination uptake	**0**	**0**	**0**	**0**	**0**	**0**	**0**	**0**	**0**	**0**	**0**	**0**	**0**	**0**	**0**	**0**
Vac_Eff_Inf	Vaccination effectiveness on infecti	**0.99**	**0.99**	**0.99**	**0.99**	**0.99**	**0.99**	**0.99**	**0.99**	**0.99**	**0.99**	**0.99**	**0.99**	**0.99**	**0.99**	**0.99**	**0.99**
f_CCT	Fraction of Cervical Cancer patients	*0.5*	*0.5*	*0.5*	*0.5*	*0.5*	*0.5*	*0.5*	*0.5*	*0.5*	*0.5*	*0.5*	*0.5*	*0.5*	*0.5*	*0.5*	*0.5*
CCT_D	Fraction of treated Cervical Cancer	*0.35*	*0.35*	*0.35*	*0.35*	*0.35*	*0.35*	*0.35*	*0.35*	*0.35*	*0.35*	*0.35*	*0.35*	*0.35*	*0.35*	*0.35*	*0.35*
CCU_D	Fraction of untreated Cervical Canc	1	1	1	1	1	1	1	1	1	1	1	1	1	1	1	1
CCT_dur	Duration with cervical cancer treate	5	5	5	5	5	5	5	5	5	5	5	5	5	5	5	5
CCU_dur	Duration with cervical cancer untrea	5	5	5	5	5	5	5	5	5	5	5	5	5	5	5	5
Im_dur	Immunity duration (years)	10															
**Economics**																	
Rf	Financial discount rate	3.5%															
Re	effect QALY discount rate	3.5%															
CoV	Cost of Vaccination	152.00															
CoD	Cost of premature death																
CoPLL	Cost of productivity loss																
Utility	Utility healthy	1	1	1	0.99	0.98	0.97	0.96	0.95	0.93	0.91	0.89	0.87	0.84	0.81	0.78	0.75
Ut_CC	Rel Utility cervical cancer	0.62															
Ut_UT	Relative utility untreated vs treated	0.5															
CoCaseCCt	Cost per CC case treated	26250															

Bilbliography

1. Population Pyramid. Population Pyramids of Iran from 1950 to 2100 [Internet]. PopulationPyramid.net. [cited 2023 Jun 30]. Available from: https://www.populationpyramid.net/world/2023/

2. Macrotrends. Iran Birth Rate 1950-2023 [Internet]. [cited 2023 Jun 30]. Available from: https://www.macrotrends.net/countries/IRN/iran/birth-rate

3. Macrotrends. Iran Death Rate 1950-2023 [Internet]. 2023 [cited 2023 Jun 30]. Available from: https://www.macrotrends.net/countries/IRN/iran/death-rate

4. Darroch JE, Landry DJ, Oslak S. Age differences between sexual partners in the United States. Fam Plann Perspect. 1999;31:160–7.

5. Twenge JM, Sherman RA, Wells BE. Declines in Sexual Frequency among American Adults, 1989–2014. Arch Sex Behav. 2017;46:2389–401.

6. Trottier H, Mahmud S, Prado JCM, Sobrinho JS, Costa MC, Rohan TE, et al. Type-Specific Duration of Human Papillomavirus Infection: Implications for Human Papillomavirus Screening and Vaccination. The Journal of Infectious Diseases. 2008;197:1436–47.

7. NCI. HPV and Cancer [Internet]. 2019 [cited 2023 Jun 30]. Available from: https://www.cancer.gov/about-cancer/causes-prevention/risk/infectious-agents/hpv-and-cancer

8. CDC. HPV Vaccine Safety and Effectiveness | CDC [Internet]. 2022 [cited 2023 Jun 27]. Available from: https://www.cdc.gov/vaccines/vpd/hpv/hcp/safety-effectiveness.html

9. Bruni L, Albero G, Serrano B, Mena M, Collado JJ, Gómez D, Muñoz J, Bosch FX, de Sanjosé S. ICO/IARC Information Centre on HPV and Cancer. Human Papillomavirus and Related Diseases in Iran (Islamic Republic of). Summary Report 10 March 2023 [Internet]. Spain: ICO/IARC; 2023. Available from: https://hpvcentre.net/statistics/reports/IRN.pdf

10. American Cancer Society. Cancer Facts & Figures 2023. 2023 [cited 2023 Jun 30]; Available from: https://www.cancer.org/cancer/types/cervical-cancer/detection-diagnosis-staging/survival.html

11. NICE. Guide to the Methods of Technology Appraisal. London: NICE; 2004.

12. Sargazi N, Takian A, Daroudi R, Nahvijou A, Yaseri M, Ghanbari Motlagh A, et al. Cost-Benefit Analysis of Human Papillomavirus Vaccine in Iran. J Prev (2022). 2022;43:841–57.

13. Emrani Z, Akbari Sari A, Zeraati H, Olyaeemanesh A, Daroudi R. Health-related quality of life measured using the EQ-5D–5 L: population norms for the capital of Iran. Health Qual Life Outcomes. 2020;18:108.

14. Global Burden of Disease Collaborative Network. Global Burden of Disease Study 2017 (GBD 2017) Disability Weights | GHDx [Internet]. Seattle, United States of America: Institute for Health Metrics and Evaluation (IHME); 2018 [cited 2023 Jun 30]. Available from: https://ghdx.healthdata.org/record/ihme-data/gbd-2017-disability-weights
